# Temperature alters the physiological response of spiny lobsters under predation risk

**DOI:** 10.1093/conphys/coaa065

**Published:** 2020-08-25

**Authors:** Felipe A Briceño, Quinn P Fitzgibbon, Elias T Polymeropoulos, Iván A Hinojosa, Gretta T Pecl

**Affiliations:** 1 Institute for Marine and Antarctic Studies (IMAS), University of Tasmania, Hobart, Tasmania 7001, Australia; 2 Crustacean Ecophysiology Laboratory, Universidad Austral de Chile, Los Pinos s/n, Pelluco, Puerto Montt 5480000, Chile; 3 Millennium Nucleus for Ecology and Sustainable Management of Oceanic Islands (ESMOI), Departamento de Biología Marina, Universidad Católica del Norte, Coquimbo, 1781421, Chile; 4 Centro de Investigación en Biodiversidad y Ambientes Sustentables (CIBAS), Facultad de Ciencias, Departamento de Ecología, Universidad Católica de la Santísima Concepción, Concepción 4090541, Chile

**Keywords:** Climate change, *Jasus edwardsii*, predator–prey interaction, respiratory physiology

## Abstract

Predation risk can strongly shape prey ecological traits, with specific anti-predator responses displayed to reduce encounters with predators. Key environmental drivers, such as temperature, can profoundly modulate prey energetic costs in ectotherms, although we currently lack knowledge of how both temperature and predation risk can challenge prey physiology and ecology. Such uncertainties in predator–prey interactions are particularly relevant for marine regions experiencing rapid environmental changes due to climate change. Using the octopus (*Octopus maorum*)–spiny lobster (*Jasus edwardsii*) interaction as a predator–prey model, we examined different metabolic traits of sub adult spiny lobsters under predation risk in combination with two thermal scenarios: ‘current’ (20°C) and ‘warming’ (23°C), based on projections of sea-surface temperature under climate change. We examined lobster standard metabolic rates to define the energetic requirements at specific temperatures. Routine metabolic rates (RMRs) within a respirometer were used as a proxy of lobster activity during night and day time, and active metabolic rates, aerobic scope and excess post-exercise oxygen consumption were used to assess the energetic costs associated with escape responses (i.e. tail-flipping) in both thermal scenarios. Lobster standard metabolic rate increased at 23°C, suggesting an elevated energetic requirement (39%) compared to 20°C. Unthreatened lobsters displayed a strong circadian pattern in RMR with higher rates during the night compared with the day, which were strongly magnified at 23°C. Once exposed to predation risk, lobsters at 20°C quickly reduced their RMR by ~29%, suggesting an immobility or ‘freezing’ response to avoid predators. Conversely, lobsters acclimated to 23°C did not display such an anti-predator response. These findings suggest that warmer temperatures may induce a change to the typical immobility predation risk response of lobsters. It is hypothesized that heightened energetic maintenance requirements at higher temperatures may act to override the normal predator-risk responses under climate-change scenarios.

## Introduction

Changes in predator–prey interactions, as a function of ocean warming, are resulting in considerable challenges for biological systems, particularly in regions experiencing significant warming such as south-eastern Australia (Hobday and Pecl, 2014). The south-eastern Australian region is one the fastest-warming regions in the southern hemisphere, and projections based on A1F1 scenarios (IPCC, 2007) suggest an increase in sea surface temperature (SST) of ~+3°C by the year 2060 (IPCC, 2007; Pecl *et al*., 2009). In Tasmania, warming temperatures have facilitated increased larval survival and settlement of a habitat-modifying sea urchin (*Centrostephanus rodgersii*) resulting in the formation of urchin barrens, i.e. reef areas that have been stripped of most algae (see Ling *et al*., 2009; Johnson *et al*., 2011). This has created structural and functional changes across multiple ecosystem levels (Ling *et al*., 2009; Johnson *et al*., 2011), with uncertain implications for key ecologically and economically important species, such as the southern rock (spiny) lobster *Jasus edwardsii* (Pecl *et al*., 2009; Johnson *et al*., 2011; Hinojosa *et al*., 2015; Pecl *et al*., 2019).

The ecosystem function that lobsters provide to Tasmanian rocky reefs may also be affected by key lobster predators such as the Maori octopus (*Octopus maorum*) (Marzloff *et al*., 2016). Octopus are considered dominate natural predators of lobsters (Anderson, 1999; Mills *et al*., 2008; Mislan and Babcock, 2008) as well as predating on lobsters trapped in fishing pots (a source of mortality known as ‘depredation’, Uhlmann and Broadhurst, 2013) (Harrington *et al*., 2006; Briceño *et al*., 2015). A recent study on *J. edwardsii* suggests that octopus predation on trapped lobsters may increase with warming temperatures, indirectly affecting lobster predation on the destructive range-extending sea urchin (Marzloff *et al*., 2016). Moreover, changes in octopus abundance and distribution are already occurring in the south-eastern Australian region(e.g. Ramos *et al*., 2014), as cephalopods are quickly responsive to temperature changes (Robin *et al*., 2014; Rodhouse *et al*., 2014; Doubleday *et al*., 2016).

Lobsters can display a strong circadian pattern in foraging behaviour, being active at night and remaining inside shelters during the day (MacDiarmid *et al*., 1991). Such a pattern, however, can be modified under predation risk (Weiss *et al*., 2008). Lobsters are able to detect predator chemical cues (known as ‘kairomones’; see Ferrari *et al*., 2010), allowing prey individuals to detect and therefore potentially avoid predators from a distance (Childress and Jury, 2006). In particular, lobsters can alter key behavioural traits once exposed to octopus presence, affecting lobster habitat selection and increasing sheltering behaviour (Berger and Butler, 2001; Mills *et al*., 2008; Butler and Lear, 2009). Recent studies have demonstrated that octopus presence can also alter *J. edwardsii* physiology: threatened individuals reduced their metabolic rates for around 3 h after being exposed to octopus kairomone (Briceño *et al*., 2018). Such a match between physiological (e.g. reduced metabolic rate) and behavioural (e.g. inactivity or immobility) traits has been reported in different taxa (Holopainen *et al*., 1997; Cooke *et al*., 2003; Steiner and Van Buskirk 2009; Krams *et al*., 2013; Okuyama, 2015; Kenison and Williams, 2018; Paul *et al*., 2018). Even though such immobility (or ‘freezing’, Smith, 1989) behaviour by prey individuals under predation risk may be energetically low-cost compared with active escaping responses (e.g. Briceño *et al*., 2018), it is unclear how both anti-predator strategies will respond to environmental variability due to climate change, e.g. warming of SST.

The trade-off between foraging activity and predation risk can be strongly modulated by environmental stressors such as temperature (Killen *et al*., 2013; Culler *et al*., 2014; Matassa and Trussel 2014; Miller *et al*., 2014; Klein *et al*., 2018), as the energetic demands in ectotherms are largely influenced by temperature (Angilletta *et al*., 2003; Dell *et al*., 2014). Under warming temperatures, the amount of energy required by prey for maintenance or survival is expected to increase, with implications for individual energy reserves (Hawlena and Schmitz, 2010; Schmitz *et al*., 2010). Moreover, recent studies suggest that temperature-dependent growth of spiny lobsters was also restricted by their capacity to consume sufficient food to meet the increased energetic demands at high temperatures (Fitzgibbon *et al*., 2017).

The oxygen- and capacity-limited thermal tolerance (OCLTT) hypothesis (Pörtner, 2010) states that a mismatch between oxygen demand and the limited capacity of oxygen supply to tissues restricts the thermal tolerance windows for marine organisms (Pörtner, 2010). The optimal thermal window for a species therefore lies between the lower and upper pejus temperatures (the Latin ‘pejus’ means ‘getting worse’), and outside this window a reduction in the oxygen levels in the body fluids occurs (hypoxemia), which decreases the aerobic scope for animal performance (Pörtner, 2010). Aerobic scope (AS) represents the amount of energy available to perform aerobic metabolism above maintenance requirements, a proxy used to assess whole-animal performance and fitness in aquatic organisms (Fry, 1947; Pörtner, 2010). In a predator–prey context, the prey escaping response implies a considerable energetic cost for aquatic prey as typically anaerobic metabolism results (Marras *et al*., 2011; Killen *et al*., 2015), requiring long-term recovery periods, which can last hours in some spiny lobsters (e.g. 8–12 h in *Sagmariasus verreauxi*, Jensen *et al*., 2014). Excess post-exercise oxygen consumption (EPOC) is used as a proxy of recovery periods after anaerobic activity and is characterized by a rise in aerobic metabolism. During the recovery period, a prey individual will use a proportion of its AS until recovery is complete, restricting other oxygen-consuming physiological functions (Killen *et al*., 2015). Previous studies have revealed that EPOC can be intensified by elevated temperatures in aquatic organisms (e.g. Fitzgibbon *et al*., 2014; Killen *et al*., 2015), although examinations linking physiological and behavioural responses under thermal scenarios are lacking, currently limiting our understanding of climate-change impacts on predator–prey relationships (Draper and Weissburg, 2019).

Considering previous information on *J. edwardsii* physiology under predation risk (Briceño *et al*., 2018), we hypothesize that lobster sub adult routine metabolism (a proxy of individual activity) may be decreased under predation risk (kairomone exposure), resulting in a reduction of activity (or immobility) as a commonly known anti-predatory response in aquatic crustaceans. However, warming waters will impose an elevated energetic maintenance requirement, which may increase the need for foraging activity and consequently change the predation risk response. To test this hypothesis, we examined different metabolic traits in sub adult *J. edwardsii* related to predator–prey interactions under thermal scenarios based on projected temperatures for the south-eastern Australian region (Pecl *et al*., 2009). Here, two scenarios were defined: current (20°C) and warming (23°C) in combination with presence/absence of predator risk. First, energetic maintenance requirements were evaluated at both temperatures by examining standard metabolic rate (SMR) in order to define the energetic ‘baseline’ of sub adults. Second, we examined routine metabolic rates (RMRs) as a proxy of lobster activity within the respirometry chambers after octopus olfactory cue (kairomones) exposure to test changes in physiological responses (such as immobility) under the two thermal scenarios. Third, lobster-escape responses such as tail-flipping were examined by active metabolic rates (AMRs) and EPOC.

## Methods

### Animals

Subadult *J. edwardsii* were collected as pueruli (first benthic post-larval stage) from the wild in southern and eastern Tasmania and reared in the facilities of the Institute for Marine and Antarctic Studies (Taroona) between 2011 and 2014. A total of 100 inter-moult individuals (~50–60 mm CL) were randomly selected and grouped for 3 weeks in a large tank (1900 L) (holding tank) between October and November (2014). The tank was supplied with flow-through water, where water temperature ranged between 15 and 17°C and the salinity was ~34 PSU. Over this period, lobsters were fed with live mussels (*Mytilus galloprovincialis*) every 2 days as suggested by Simon and James (2007). Lobsters were kept in a natural light cycle of 13–15-h day length over this period.

Two male octopuses (*Octopus maorum*) (6–8 kg) were used to create a nocturnal predation-risk scenario. These individuals were collected from a scientific reserve with an area of ca. 1 km^2^ at Crayfish Point near Taroona in Tasmania, Australia (42°57.2′S: 147°21.2′E). Octopuses were individually placed in 800-L circular tanks provided with artificial shelters and covered with black mesh to suppress escaping. Individuals were fed with prawns (*Fenneropenaeus merguiensis*) daily to satiation (*ad libitum*). Environmental conditions (temperature, salinity and photoperiod) were the same as described for lobsters above. Octopus collection, maintenance and handling were conducted under the University of Tasmania Animal Ethics Committee, permit approval no. A0013584.

## Experimental design

### Thermal scenarios

The current maximum water temperature over summer in northern Tasmania (20°C) was used as a proxy for the maximum temperatures commonly experienced by *J. edwardsii* in Tasmania. Considering this temperature as a base line, SST projections under the IPCC-A1F1 scenario (IPCC, 2007) for the south-east Australian region for the year 2060 (+ 3°C) resulted in 23°C. Hence, these thermal scenarios were defined as ‘current’ (20°C) and ‘warming’ scenarios (23°C).

A total of 48 lobsters were randomly selected from the inter-moult lobster population previously described, with sex and body weight individually recorded. Animals were acclimated to these thermal scenarios between January and March 2015 for between 7 and 14 days, a suitable period to achieve metabolic acclimation in lobsters (e.g. *Sagmariasus verreauxi*, Fitzgibbon *et al*., 2014).

In order to avoid prolonged acclimation periods and variability among individuals, lobster acclimation was conducted in four consecutive rounds (‘acclimation rounds’) including 12 sub adults per round. Acclimation rounds were conducted in four 57-L rectangular tanks at a density of three individuals per tank. Tanks were provided with shelters built with oyster mesh (5 mm mesh size) to reduce agonistic behaviour in *J. edwardsii* sub adults (Carter *et al*., 2015). In addition, these tanks were supplied with water from an open-flow system from two head tanks (450 L each one) where the experimental temperatures were achieved via two immersion heaters (8.33 A, 2000 W). The tanks were supplied with a continual air supply through air stones, and water volume was exchanged 3.5 times per hour, keeping levels of dissolved oxygen at or near saturation (>90%) and ammonia levels > 1.0 mg L^−1^. Temperature was logged every 2 h.

Lobsters were fed with fresh mussels (half-shell per lobster) every second day during each acclimation round. Moulting individuals occurred at very low numbers (<5%) over the acclimation period, and they were excluded from respirometry measurements given profound physiological changes in *J. edwardsii* associated with moulting (Simon *et al*., 2015). Additionally, before any respirometry measurements were taken, lobsters were fasted for 72 h to standardise the post-prandial state among individuals (Jensen *et al*., 2013).

Octopus were randomly selected and acclimated at the same experimental temperatures for 72 h before lobster respirometry was undertaken. This allowed a match between thermal and predation risk scenarios, as well as reduced thermal stress in the octopus. No food was provided over this period to avoid predator diet cues (faeces) as a confounding factor in the predation risk experiments (e.g. Ferrari *et al*.,2010). Octopus acclimation was performed in a 200-L circular tank (shelters and covering mesh provided) supplied with water from a circular tank (800 L) in which a heater was installed (8.33 A, 2000 W). Animals were gradually acclimated from normal temperature to the thermal scenarios over 72 h using a warming rate of 0.5–1°C per day.

### General set-up for respirometry

Metabolic states analysed here were calculated from measurements of oxygen consumption or metabolic rates (*ṀO*_2_) and were conducted with an intermittent-flow respirometry system consisting of two 3.55-L respirometric chambers (radius: 48 mm; length: 480 mm) described by Jensen *et al*. (2013). Here, each trial consisted of two lobsters placed individually in each of the respirometry chambers (see supplementary video) and simultaneously exposed to thermal and predation risk scenarios as described below. Each chamber had oyster mesh (5-mm mesh size) fitted to the lower section to provide a tractional surface as suggested for lobster respirometry (e.g. Jensen *et al*., 2013). Animals were able to move along the chamber, and an oyster mesh cylinder (15 cm × 7 cm) was included within the chamber to promote sheltering behaviour.

Dissolved oxygen was measured every 10 s by a luminescent dissolved oxygen optode (Hach LDP, HQ40d, Hach company, USA). Two submersible aquarium pumps (Quiet One 1200) were connected to each chamber. One pump was used for mixing the water inside the chamber and delivery of water past the oxygen optode at a rate of 1.0 exchange min^−1^ (3.55 L min^−1^). The flushing cycle was performed every 5 min by connecting the pump to a digital timer (DRT-1, Sentinel, China). The closing or sealing cycle of the chamber was performed every 10 min. This resulted in measurements of *ṀO*_2_ every 15 min. Briefly, lobsters were placed into the respirometer around midday and *ṀO*_2_ was continually monitored for 26 h resulting in 104 individual *ṀO*_2_ measurements. The first 6 h were considered as an acclimation period, which were not included in the analyses. Respirometric chambers were carefully rinsed with freshwater after each trial and sterilized with a chlorine solution every two trials. In addition, oxygen saturation was kept above 90% (e.g. Jensen *et al*., 2013; Fitzgibbon *et al*., 2014) and background oxygen consumption was measured in empty chambers after each trial for 3–6 h. After respiratory measurements, lobsters were removed from the chambers and their wet weight recorded using a digital scales (±0.01 g). Animals were returned to the acclimation tanks until the acclimation round was finished. Over this period, animals were constantly observed and moulted individuals were removed from the analysis. Respirometry was conducted under a natural light cycle, with the nocturnal period between 20:00 and 8:00, under a natural light cycle.

### Predation risk scenarios

**Figure 1:**
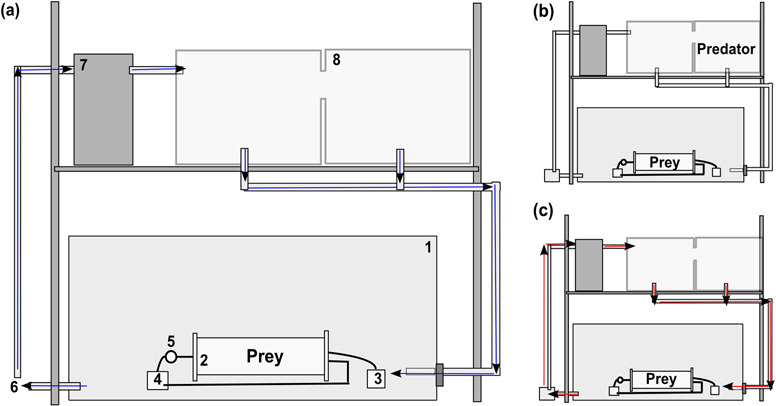
Experimental set-up used on sub adult *J. edwardsii* (prey) respirometry under exposure of kairomones from octopus odour (predator). The set-up consisted of (1) bath reservoir (treatment tank), (2) two respirometric chambers, (3) flushing pump (which pumps water from the bath into the chambers), (4) mixing pump, (5) oxygen probe, (6) recirculating pump, (7) heater/chiller unit and (8) conditioning tank. Blue arrows show direction of water circulation throughout the system without kairomones. Red arrows show the direction of water with kairomones from the conditioning tank to the bath. Details on experimental set-up are provided in Supplementary Information.

Respirometry trials were carried out in a recirculating water system designed to expose lobsters to octopus kairomones (‘cues’), which were used as a proxy for predation risk (Fig. 1). The system consisted of a conditioning tank (200 L) where an octopus was placed and which could be connected to a 455-L treatment tank (‘bath’) where an intermittent respirometry system was immersed.

Nocturnal predation risk scenarios were simulated by the inclusion of kairomones from octopus during lobster respirometry. Two predation risk scenarios resulted from the absence (‘− Risk’) or presence (‘+ Risk’) of such predator cues, considered as treatment and control trials, respectively. In addition, these predation risk scenarios were randomly undertaken in combination with the thermal scenarios previously described. A protocol describing the steps performed for the generation and exposure of kairomones is shown in Supplementary Table 1. Importantly, the same protocol was applied for control trials, differing only in the presence of octopus in the conditioning tank.

The total number of lobsters used in this study was slightly reduced from the original experimental design (*n* = 48) given some lobster mortalities (i.e. individuals escaping from the experimental system) and some moulting occurred during acclimation rounds. This resulted in an unbalanced design with a total of 35 individuals finally used for this experiment, which is summarized in Table 1. Lobster body weight did not differ among treatment groups according to an analysis of variance (ANOVA) (*F* = 0.232; *P* = 0.633).

### Metabolic states

From *ṀO*_2_ measurements previously described, we examined different metabolic states from lobsters under thermal and predation risk scenarios, including standard metabolic rates (SMRs), routine metabolic rates (RMRs), excess post-excercise oxygen consumption (EPOC) and active metabolic rates (AMRs). 

### Standard metabolic rates at different temperatures (SMR)

SMR is defined as the minimal maintenance or resting metabolic rate of an unstressed, post-absorptive, non-reproductive and inactive individual while in its resting phase, measured at a specific temperature (Fry, 1971; Careau *et al*., 2015). We used SMR as a proxy to define energetic requirements from lobsters without predation risk (as predator cues may modify the unstressed condition necessary for determining SMR) and under both thermal scenarios (20 and 23°C). Given the limited information on standard metabolism in sub adult *J. edwardsii* across temperatures (Crear and Forteath, 2000), we further examined the SMR at two lower temperatures, 14°C (*n* = 3) and 17°C (*n* = 4), in order to achieve a better understanding of the relationship between SMR and temperature. SMR was calculated following Fitzgibbon *et al*. (2014) as the mean of the lowest 10% of all values exclusively for treatments without predation risk (control trials) under the conditions mentioned above.

**Table 1:** Summary of replicates per treatment from thermal and predation risk scenarios used in sub adults *J. edwardsii* (50–60 mm of carapace length)

***Predation risk/thermal scenarios***	***Current scenario (20°C)***	***Warming scenario (23°C)***
***Absence (− risk)***	*n* = 10 (79.6 ± 11.8 g) 80% female	*n* = 12 (76.2 ± 16.0 g) 58% female
***Presence (+ risk)***	*n* = 7 (76.0 ± 9.2 g) 57% female	*n* = 8 (77.2 ± 7.9 g) 25% female

Mean body weight and variability (± se) is included

**Table 2:** Exponential growth regression describing the relationship between temperature (14–23°C) and standard metabolic rate of *J. edwardsii* sub adults (body weight = 79 g ± 13 g).

***Coefficient***	***Estimate***	***Std. error***	***Df***	***t value***	***P value***
***Fixed effect***					
***a (intercept)***	0.009	0.238	27	−19.157	<0.001
***b (temperature)***	0.071	0.012	27	6.108	<0.001
					
***Random effect***	***Intercept***	***Residual***			
***Std***. ***dev***	0.173	0.065			

**Figure 2:**
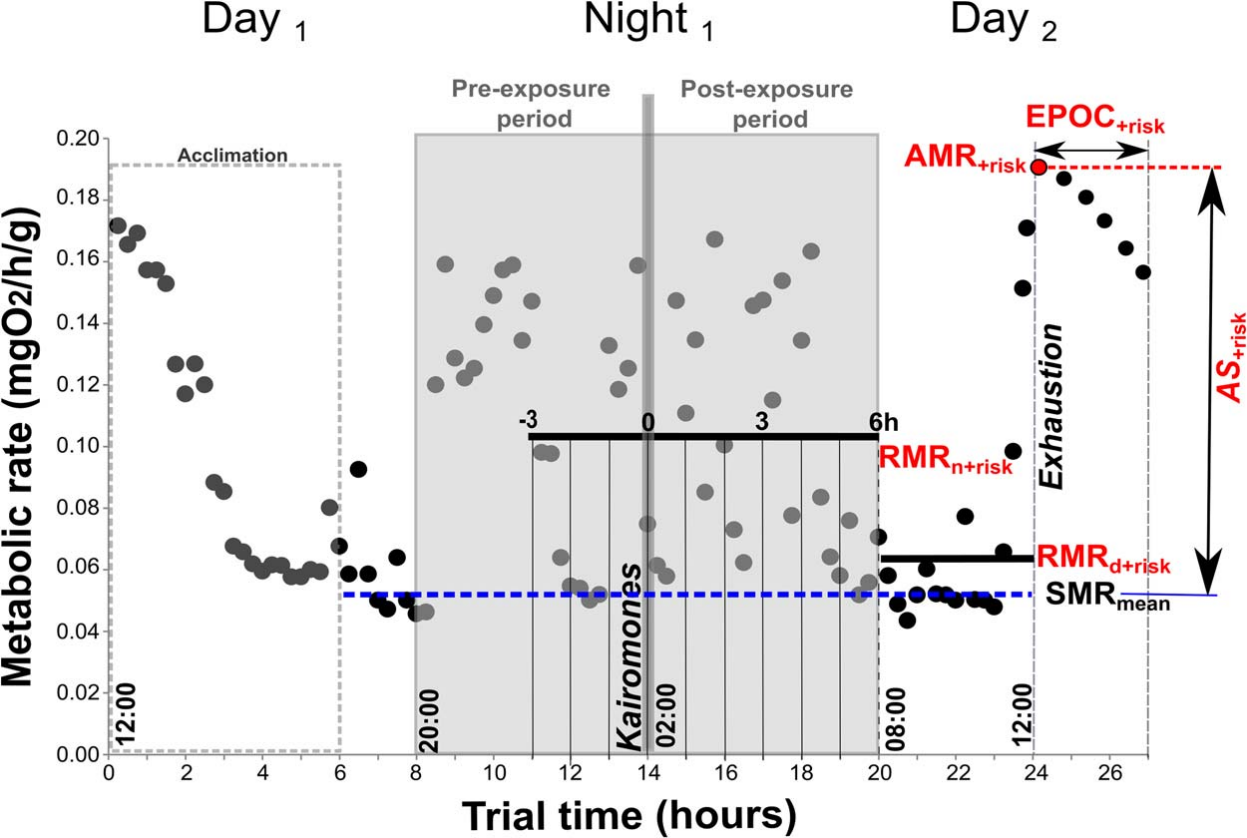
Example of a measurement of metabolic rates at 23°C under predation risk (‘+ risk’). We examined the following metabolic states: the routine metabolic rate (RMR), the standard metabolic rate (SMR) and the active metabolic rate (AMR). Additionally, the excess post-excercise oxygen consumption (EPOC) and aerobic scope (AS) are shown. Each dot represents an average measurement of metabolic rate over a period of 15 minutes. RMR was calculated as the mean value per hour, examined during the night time (‘RMRn’) and day time (‘RMRd’). SMR was estimated as the mean value of the lowest 10% of the measurements and only calculated for lobsters without predation risk. ‘AS + risk’ was calculated using mean values of SMR (SMRmean) for each temperature. AMR and EPOC were obtained after exhaustion as specified by the dashed vertical line. Additionally, the dashed box represents the acclimation period (6 hours) and the grey box represents the nocturnal period defined between 20:00 and 08:00. The predation risk scenario was generated by exposing the lobsters to octopus (*Octopus maorum*) cues (kairomones) performed approximately at 02:00 as illustrated by the vertical grey line. In the no predation risk scenario, this procedure was performed with sea water only.

### Routine metabolic rates under predation risk and temperature (RMRs)

RMR relates to the *MO_2_* post-absorptive, non-reproductive and undisturbed animal that also includes the costs of spontaneous activity and the maintenance of posture and equilibrium (Fry, 1971; Careau *et al*., 2015). In our study, we used RMR to measure lobster activity within the respirometry chambers. The effect of predation risk and thermal acclimation scenarios on lobsters was examined for nocturnal and diurnal RMR following Briceño *et al*. (2018). The nocturnal RMR (RMRn) was used as a proxy of nocturnal activity pre and post treatment in relation to kairomone exposure. RMRn was calculated as the mean hourly *ṀO*_2_, examined between 3 h before and 6 h after kairomone exposure (KE) (the period between 2:00 and 8:00, referred as ‘RMRn+risk’). In addition, diurnal activity was estimated via diurnal RMR (RMRd) examined over the period between 08:00 and 12:00, resulting in ‘RMRd-risk’ and ‘RMRd+risk’ for sub adults under the absence and presence of predation risk, respectively.

After 24 h of the initiation of each trial (approximately between 13:00 and 14:00), each animal was removed from the chamber and swum until exhaustion by manually encouraging the lobster to swim following the method described by Fitzgibbon *et al*. (2014). This chasing protocol was performed on animals in both predation risk scenarios. Animals were exercised in a circular tank (100 L) for ~3 min until lobsters became exhausted and non-responsive to stimuli by hand. Lobsters were immediately replaced in the respirometer and measurements taken to estimate the EPOC. EPOC was examined at 15, 30, 45 and 60 min post-exhaustion. The resulting EPOC for animals under predation risk was referred to as ‘EPOC+risk’. The AMR was defined as the maximum EPOC, which generally occurred at the first recording after exhaustive exercise (Jensen *et al*. 2014; Fitzgibbon *et al*. 2014). Under the predation risk scenario, this metabolic rate was referred to as ‘AMR+risk’. Finally, the AS was calculated as the difference between AMR and SMR, which was calculated differently for each predation risk scenario. The AS under predation risk (‘AS+risk’) was calculated using ‘AMR+risk’ from each individual but using mean values of SMR (SMRmean) (Fig. 2) for each temperature (20 and 23°C).

### Analysis

Lobster *ṀO*_2_ and background respiration were determined by applying linear regressions to the rate of decline of dissolved oxygen concentration during the respirometer closed (non-flushing) cycle. *ṀO*_2_ was expressed in mg O_2_ h^−1^ g^−1^ after the subtraction of background respiration.

We applied generalized linear mixed models (GLMMs) to examine the effect of temperature and predation risk on lobster standard, routine and active metabolisms. While each of these metabolisms was examined by different variables used as the fixed term in the GLMMs, we incorporated the ‘individual’ as a random term. In doing so, the lack of independency from any pseudo-replication that may have occurred (because each trial was run with two lobsters simultaneously) could be solved by using the random term (Zuur *et al*., 2009). A similar approach has been applied in previous studies examining lobster physiology under predation risk (see Briceño *et al*., 2018). All GLMMs included the same random term as specified.

To examine the effect of temperature levels (14, 17, 20 and 23°C) on SMR, a GLMM using temperature as a fixed factor and log-SMR as an explanatory variable was used. Thus, the relationship between temperature and SMR was further examined by fitting an exponential curve. Given the imbalance between replicates over the experimental temperatures, a Type II ANOVA was undertaken to test significance of temperature.

The effect of predation risk and temperature on lobster routine metabolism was analyzed for night and day (‘period’) after KE. The interaction period*predation risk was examined as a fixed factor in the GLMMs, independently for each thermal scenario. This modelling approach allowed us to better examine variability exclusively associated with the interaction term (e.g. RMR changes during day and night under predation risk); otherwise, GLMM outcomes were masked by the strong effect of temperature on lobster metabolism. The significance of factors was further examined by analysis of variance (ANOVA, Type II) with significant differences identified by Tukey’s HSD tests for post hoc multiple comparisons.

The effect of predation risk and temperature on AMR and AS was explored by Type II ANOVA to examine the interaction predation risk * temperature. EPOC was compared with pre-exhaustion RMRd by two-tailed independent t-tests. Significance levels were set at *P* < 0.05, and all analyses were performed in R (R Development Core Team 2014), using packages ‘lme4’ for the GLMMs, ‘car’ for ANOVA and ‘ls means’ for Tukey’s HSD tests.

## Results

### The effect of temperature on standard metabolism

**Figure 3:**
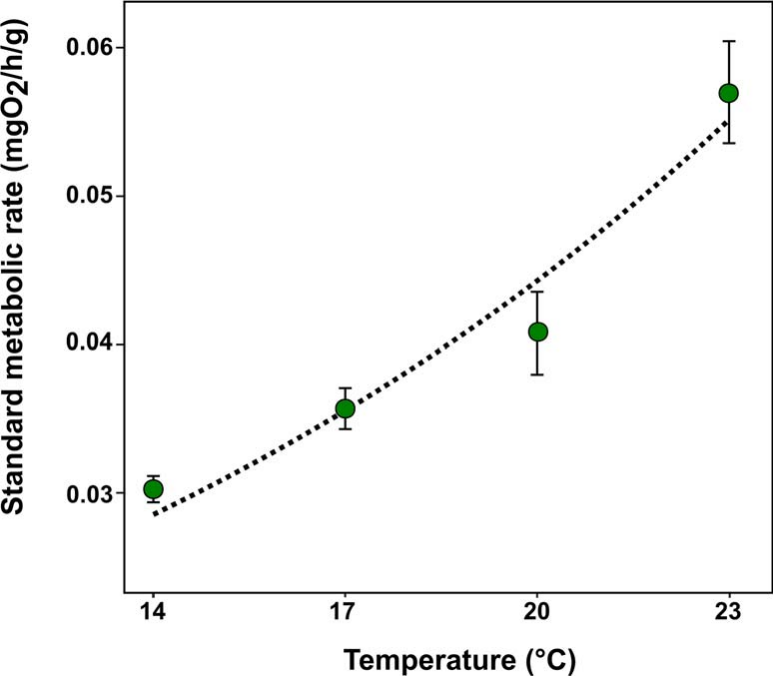
(a) Exponential growth in mean values of standard metabolic rate at 14°C (*n* = 3), 17°C (*n* = 4), 20°C (*n* = 10) and 23°C (*n* = 10) for sub adults *J. edwardsii*. Vertical bars represent standard errors. Dashed line represents exponential fitting. Details on exponential fitting are provided in Table 2.

The standard metabolic rate of lobsters exponentially increased between 14 and 23°C (*χ*^2^ = 37.304, df = 1, *P* < 0.001) (Fig. 3). Coefficients from the SMR–temperature relationship are shown in Table 2. In particular, SMR increased around 39% between 20 and 23°C with a relatively similar variability among individuals (coefficient of variance, CV = 21%).

**Figure 4:**
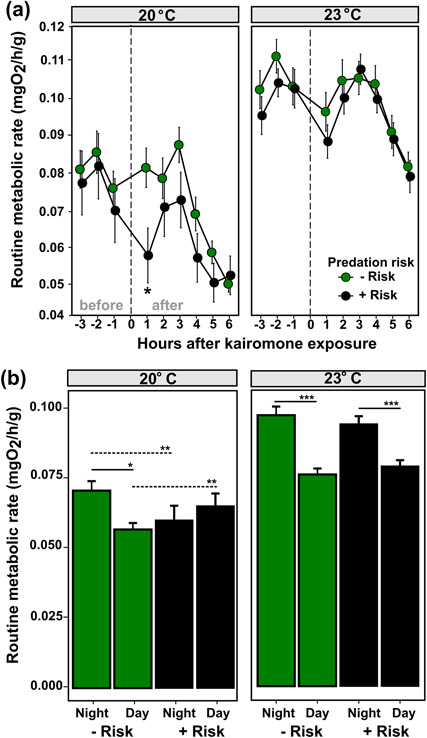
(a) Temporal changes (hourly) in RMRs for sub adult *J. edwardsii* after kairomone exposure representing absence (– risk) and presence (+ risk) of predation risk at two temperatures: 20°C and 23°C. Dashed vertical lines represent the time (approximately at 02:00 am) when animals were exposed with kairomones, and the 6-hour period after the exposure until around 8:00. Significance between treatments (ANOVA test, type II) (– risk vs + risk) is expressed by an asterisk. Vertical bars represent individual variability (mean + 1 SE). (b) Mean routine metabolic rates at day and night for sub adults under conditions of absence (– risk) or presence (+ risk) of predation risk at ambient (20°C) or projected warming (23°C) temperatures. Horizontal bars represent intra (solid) and intertreatment (dashed) differences, with asterisks specifying the level of significance: ^*^ <0.05, ^**^ <0.01; ^***^ <0.001.

### Temporal changes in routine nocturnal metabolism after KE

Overall, lobster RMR at 20°C was 41.9% lower than at 23°C, regardless of risk scenario. RMR 1 to 3 h before KE (the pre-exposure period) was similar between predation risk scenarios at 20°C (*χ*^2^ = 0.049, df = 1, *P* = 0.824) and 23°C (*χ*^2^ = 0.306, df = 1, *P* = 0.580) (Fig. 4a). Conspicuous temporal changes in RMRn after KE were found at 20°C, but not at 23°C, resulting in the following three main periods: (1) a quick reduction in RMRn+risk between the pre-treatment period and the first hour after KE at 20°C (χ^2^ = 4.8012, df = 1, *P* < 0.05). This decrease in RMRn+risk resulted in a reduction in metabolic rate of 29% (or a difference of 0.024 mgO_2_ h^−1^ g^−1^) compared to control animals suggesting an acute response at this temperature. (2) An increase in RMRn+risk between 1 and 3 h after octopus kairomone exposure observed at both temperatures without any difference between predation risk scenarios. (3) A decrease in RMR between 3 and 6 h after KE observed at both temperatures, independent of the predation risk scenario. Nevertheless, the rate of decline was slightly more in RMRn+risk than RMRn-risk at 20°C according to the interaction predation risk * hour (*χ*^2^ = 3.357, df = 1, *P* = 0.067).

### Mean nocturnal and diurnal RMR under predation risk and temperature

At 20°C, lobsters showed a difference in mean RMR between nocturnal and diurnal periods (interaction risk * period, *χ*^2^ = 7.089, df = 1, *P* < 0.01) (Fig. 4b). For example, mean RMRn+risk was on average 17% lower than mean RMRn-risk, and mean RMRd+risk was 14% higher than mean RMRd-risk. Interestingly, nocturnal routine metabolism under predation risk was not significantly different from the diurnal metabolism of animals in the absence of risk. At 23°C, lobsters demonstrated the same pattern as controls reducing (16%) routine metabolism during the day, but this was not significantly different between predation risk scenarios (*χ*^2^ = 1.324, df = 1, *P* = 0.249). Further details about the relationship between routine metabolism and predation risk, period and temperature are provided in Table 3. Overall, lobsters under predation risk at 20°C consumed ~29% less oxygen than unthreatened lobsters at the same temperature.

### AMR and AS under predation risk and temperature

**Figure 5:**
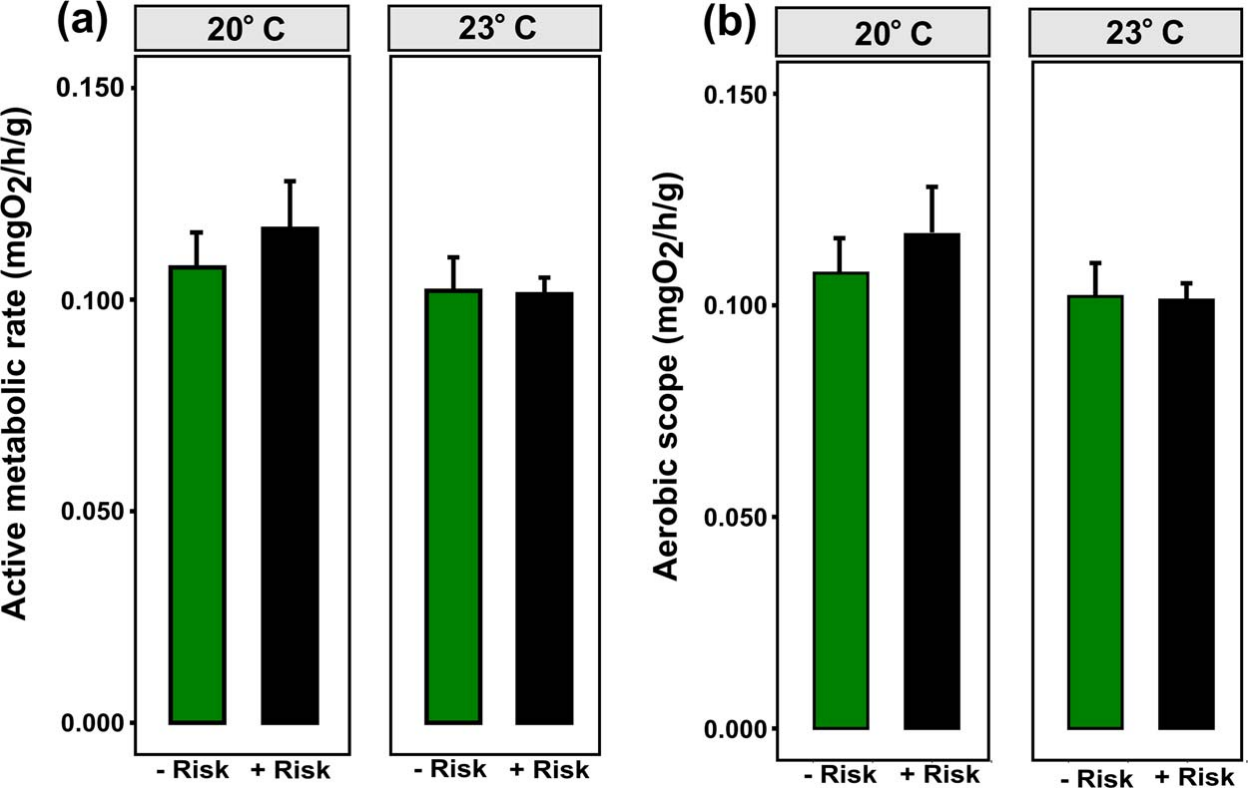
Relationship between sub adult *J. edwardsii* (a) A and temperature and (b) aerobic scope and temperature under the absence (– risk) and presence (+ risk) of predation risk. Vertical bars represent individual variability (mean + 1 SE).

Active metabolism was not affected by predation risk (*χ*^2^ = 0.202, df = 1, *P* = 0.653) or by temperature (*χ*^2^ = 0.611, df = 1, *P* = 0.435), and the interaction of both factors was also not significant (*χ*^2^ = 0.351, df = 1, *P* = 0.553) (Fig. 5a). These results indicate that, independent of the temperature and predation risk, lobsters consumed a similar amount of oxygen after exhaustion. There was an inverse relationship between the AS and the temperature for both predation risk scenarios, resulting in an elevated AS at 20°C compared to 23°C (Fig. 5b). Temperature affected AS (*χ*^2^ = 5.41; df = 1; *P* < 0.05), independently of predation risk (*χ*^2^ = 1.817, df = 1, *P* = 0. 178). Individuals under predation risk showed a drop of 0.0159 (mgO_2_ h^−1^ g^−1^) between 20 and 23°C. Furthermore, variability among individuals was higher at 20°C (25%) than at 23°C (11%).

### The effect of predation risk and temperature on the EPOC

**Figure 6:**
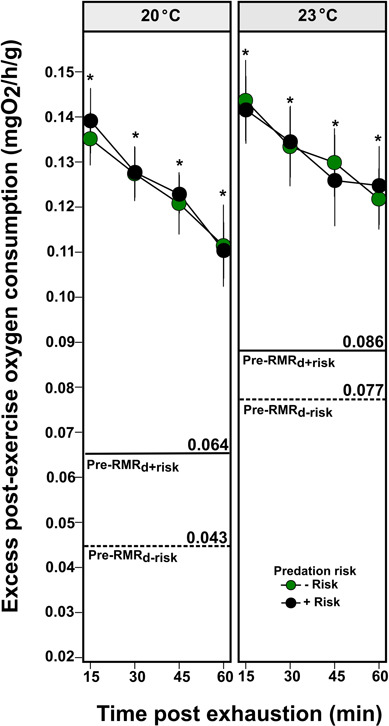
Mean EPOC rate of *J. edwardsii* sub adults at 20°C and 23°C at different time post-exhaustion (min). Horizontal lines represent the pre-exhaustion diurnal RMRs (Pre-RMRd) for individuals under absence (dashed line; ‘Pre-RMRd-risk’) and presence of predation risk (solid line; ‘Pre- RMRd+risk’). Asterisks (*) indicate significant differences from a two-tailed independent t-test (p < 0.05) between pre-exhaustion RMR and EPOC. Vertical bars represent individual variability (mean ± 1 se).

There was no difference in EPOC between predation risk levels at 20°C (*χ*^2^ = 0.077, df = 1, *P* = 0.782) or at 23°C (*χ*^2^ = 0.020; df = 1, *P* = 0.887) (Fig. 6). However, temperature affected EPOC (*χ*^2^ = 12.225; df = 1, *P* < 0.001), although there was no difference in the temporal patterns in EPOC between temperatures (interaction time * temperature) (*χ*^2^ = 0.569; df = 1; *P* = 0.450). Furthermore, EPOC at 20 and 23°C did not return to pre-exhaustion routine metabolic levels during the examination period (within 60 min after exhaustion).

**Table 3:** Generalized linear mixed model (GLMM) outcomes to test the effect of predation risk: ‘+ Risk’ (presence of predator kairomones) and period (night and day) at 20°C (a) and 23°C (b) on RMRs in *J. edwardsii* sub adult.

**GLMM at 20°C**					
***Fixed effect***	***Coefficient***	***Std. error***	***DF***	***t value***	***P value***
***Intercept***	0.085	0.009	15	9.35	< 0.01
***+ Risk***	−0.028	0.014	15	−1.99	0.0641
***Period***	−0.014	0.004	15	−3.28	< 0.01
Period * + Risk	0.018	0.007	15	2.662	< 0.05
					
***Random effect***	***Intercept***	***Residual***			
***Std. dev***	0.019	0.010			

**GLMM at 23°C**					
***Fixed effect***	***Coefficient***	***Std. error***	***DF***	***t value***	***P value***
***Intercept***	0.118	0.006	18	19.069	< 0.01
***+ Risk***	−0.009	0.009	18	−0.913	0.373
***Period***	−0.021	0.003	18	−6.593	< 0.01
Period * + Risk	0.006	0.005	18	1.151	0.265
					
***Random effect***	***Intercept***	***Residual***			
***Std. dev***	0.017	0.008			

## Discussion

Our examination of the respiratory physiology of *J. edwardsii* sub adults under predation risk and temperature treatment demonstrates that animals display a different metabolic response depending on predation risk and thermal scenario. Unthreatened lobsters displayed a strong circadian pattern in routine metabolism, resulting in higher oxygen consumption rates at night. This pattern was magnified at 23°C, demonstrating an elevated routine metabolism under warming scenarios. However, under predation risk, lobsters acclimated at 20°C exhibited a rapid downregulation of routine metabolism, which was not observed at 23°C. These findings suggest that warmer temperatures may induce a change to the typical predation risk response of immobility. We hypothesize that heightened energetic maintenance requirements may act to override normal predator-risk responses under climate-change scenarios.

**Figure 7:**
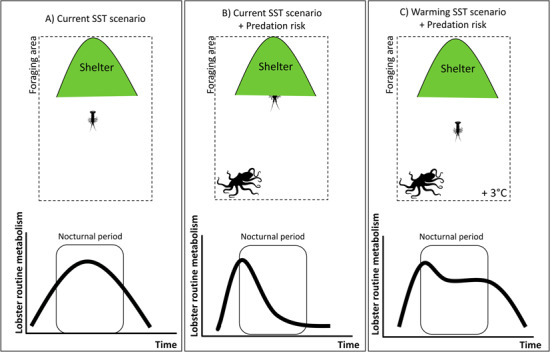
A conceptual model of decision-making (sheltering vs foraging) in sub adult *J. edwardsii* under predation risk is proposed from major outcomes of our study. Using RMRs as a proxy of lobster activity under predation risk and SST, three scenarios are explored: (A) Under current temperatures, lobster foraging occurs mainly at night, resulting in a circadian pattern in routine metabolism with a maximum nocturnal energy requirement. (B) Under predation risk and current temperatures, lobster sheltering behavior increases as an anti-predator response, involving a reduction in RMR during the night and the loss of foraging opportunity. (C) Under warming temperatures and predation risk, lobster decision-making is expected to change due to elevated energy requirements for maintenance at higher temperatures, increasing prey exposure while foraging. In this scenario, response to predation risk is overridden by the response to elevated temperatures.

### SMR and AS

The maintenance requirements, here examined by the SMR, increased exponentially between 14 and 23°C, as typically observed in ectotherms within their thermal tolerance window (Pörtner 2010). The increasing SMR between current (20°C) and warming (23°C) scenarios would suggest that sub adult lobsters would increase their maintenance requirements (approximately up to 39%) at temperatures projected for the south-eastern region of Australia by 2060. The exponential increase in SMR up to the maximum temperature examined (23°C) suggests that the upper critical temperatures were not exceeded, according to the OCLTT hypothesis (Pörtner, 2010). Previous research on the thermal physiology of *J. edwardsii* would suggest that 23°C would be very close to the critical limits for the species (Thomas *et al*., 2000). Using small juveniles (1–5 g), Thomas *et al*. (2000) reported a decreasing trend in SMR between 22 and 24°C, suggesting that the upper critical temperature limit is within this range.

In this study, lobster AS was higher at 20 than at 23°C, independent of the level of predation risk, suggesting that the upper pejus temperature was exceeded at 23°C. A previous study found that the maximum AS of sub adult *J. edwardsii* was at 13°C (Crear and Forteath, 2000), suggesting that both experimental temperatures examined in the present study (i.e. 20 and 23°C) were beyond the upper *pejus*. Beyond the *pejus* temperature, overall physiological performance diminishes due to incapacity of the cardiorespiratory system to meet increased oxygen demands associated with elevated metabolism. Nevertheless, further studies examining a broader range of temperatures in juveniles and sub adults are needed to better define thermal tolerance and associated aerobic performance in *J. edwardsii*.

### Routine metabolism under predation risk and temperature

At the current temperature scenario (20°C), lobsters exposed to octopus olfactory cues reduced their RMR by 29% during the first hour after KE. The lowering in RMRn+risk provides evidence of an immobility response (i.e. move into hiding and reducing activity), which is suggested as a predator avoidance mechanism in spiny lobsters (Buscaino *et al*., 2011; Briceño *et al*., 2018). Interestingly, the RMR of lobsters under nocturnal predation risk was similar to the diurnal RMR from controls when lobsters are typically inactive as demonstrated in *J. edwardsii* respirometry experiments (Crear and Forteath, 2000). Reduced activity is considered as a taxonomically widespread response to predation risk and an effective mechanism to avoid predation (Sih, 1985; Lima and Dill, 1990; Toscano and Monaco, 2015; Paul *et al*., 2018), commonly reported in aquatic crustaceans such as lobsters (see Hazlett 2011). Such reduction in prey activity was previously defined as ‘freezing’ in fish gobiid studies where predator cues from octopus were used (e.g. Paul *et al*., 2018). Alternatively, such immobility response could reflect a reduction in metabolites or chemical cues produced by lobsters (e.g. ‘chemical quiescent’) which may minimize the predator’s perception of the prey, as discussed previously in lobsters (e.g. Atema, 1995) and other crustaceans (e.g. Díaz and Thiel, 2004) and could be further considered in future studies.

In this study, we show that lobsters may not display an immobility response at the higher water temperatures predicted under a global warming scenario. The lack of such response at 23°C reported in this study suggests that temperature may inhibit the anti-predator mechanism, increasing exposure and therefore risk of mortality, at least under the experimental conditions tested here. As animals at warmer temperature have higher energetic requirements in order to support elevated maintenance metabolic costs, increasing activity could be expected in order to cover the required energy intake and would also therefore be associated with increasing foraging rates (Careau *et al*., 2015). This is relevant for lobster species where there appears limited capacity to regulate feeding capacity to support energetic demands at higher temperature, revealing the crucial role that feed intake plays in regulating performance at thermal extremes (Fitzgibbon *et al*., 2017). Here, projected temperatures for the region might increase the risk-taking behaviour of lobsters due to greater foraging demands and therefore expose lobsters to greater predation risk.

Another plausible explanation of the lack of immobility response at high temperature may be associated with changes in kairomone properties (e.g. protein degradation), as well as kairomone production by octopus under the warming scenario. Recent studies have shown that chemical alarm cues in tropical fish can degrade rather rapidly under natural conditions considering daily changes in water temperature, solar radiation, pH and dissolved oxygen (Chivers *et al*., 2013). The effect of temperature on the degradation rate of alarm cues may result in seasonal changes in risk perception by prey as suggested by Chivers *et al*., (2013). It is unclear whether outcomes from our study were affected by the temperature effect on kairomone properties, as well as on lobster sensory capacity, which must be considered in future examinations using the current predator-prey model. Global warming and other long-term environmental stressors (e.g. acidification) may disrupt signalling processes (e.g. signal transmission and reception) that mediate aquatic predator–prey interactions, although the lack of underlying mechanisms has strongly limited our understanding on potential impacts to marine ecosystems (Draper and Weissburg, 2019). Alternatively, the lack of immobility response in the warming scenario may have been a result of a low production of octopus kairomone. In our study, octopus individuals were acclimated at the same thermal scenarios as lobsters. Nevertheless, it is unknown whether *O. maorum* metabolism was altered under the temperatures tested as the thermal tolerance of this species has not been examined. However, under such a hypothesis it is assumed that octopus metabolism is directly linked to kairomone production. Further research is thus needed given the strong relationship between temperature and metabolism in cephalopods (Moltschaniwskyj and Carter, 2010), especially because recent studies have demonstrated a climate-driven change in distribution in some octopus species (*O. tetricus*) in the south-eastern Australian region (Ramos *et al*., 2014; Ramos *et al*., 2018).

Routine metabolism during the day (RMRd) was higher in predator risk-exposed animals than in controls at 20°C. This metabolic response could reflect the need to compensate the nocturnal reduction of activity from the immobility response, although more research is needed to test such a hypothesis. It is expected that prey individuals might need to compensate the loss of foraging opportunities during the night by additional foraging during the day in order to supply enough food to satisfy energetic maintenance costs (Careau *et al*., 2015). Consequently, a potential change in lobster foraging patterns may imply a lower risk of predation by nocturnal predators such as octopus, but a higher predation risk from diurnal predators such as fishes (Mills *et al*., 2008). Future investigation using this predator–prey model should examine changes in foraging activity after predatory exposure during night and day periods.

The immobility response of sub adult lobsters in the present study was more acute (over 1 h) than in adults (3 h), although the reduction of RMR was relatively similar (29%) to observations in adult *J. edwardsii* (31.4%) (Briceño *et al*., 2018). These differences could be attributable to intrinsic factors such as body size, body condition, sex and age, which can affect the way that prey perceive predation risk (Ferrari *et al*., 2010). For instance, studies in juvenile lobsters (*Homarus americanus*) have demonstrated that lobster size matters in sheltering/foraging behaviour, with smaller juveniles showing a stronger sheltering response than larger individuals (Wahle, 1992). Thus, some spiny lobsters (*Panulirus interruptus*) can select shelters more closely scaled to their body size in areas under high predation pressure (Loflen and Hoven, 2010). In addition, it is unclear if lobster sex could have played a role in our results. Studies have revealed that predation risk is often most pronounced for certain age and/or size classes and differs between sexes in fish, resulting in morphological anti-predator plasticity (Picklington and Dill, 1995; Meuthen *et al*., 2019). However, it is likely that the influence of sex would be negligible as lobsters were not mature. The size at maturity for this species and region is 75–80 mm CL (see Gardner *et al*., 2006).

Spiny lobsters undergo changes in social behaviours (e.g. social aggregations) throughout their ontogeny (Childress and Herrnkind, 1996; Childress, 2007). Lobsters were isolated in respirometry chambers, eliminating some anti-predatory responses such as conspecific aggregations otherwise likely observed in nature. Hence, outcomes from this study may better describe solitary lobsters, which could metabolically differ from aggregative formations. As reported for *J. edwardsii* (Butler *et al*., 1999), spiny lobsters frequently form aggregations in shelters during juvenile and sub adult stages compared to early benthic stages (e.g. post-pueruli). Additionally, sub adults can display a distinct aggregative response to chemical cues from conspecifics, especially from large lobsters, which minimizes predation risk (Butler *et al*., 1999). For example, large lobsters generally range freely in comparison with juveniles and sub adults that remain within a refuge for longer as reported in *J. edwardsii* (Butler *et al*., 1999). This is an important anti-predator strategy in young lobsters although such aggregative behaviour could have a trade-off as high competition for limited dens may occur (Butler *et al*., 1999). Additionally, experience also affects how prey individuals respond to predators (Ferrari *et al*., 2010). For instance, predator-experienced individuals are generally more responsive to predator odour compared to predator-naïve individuals (Ferrari *et al*., 2010). Lobsters used in this study were raised from early benthic stages (pueruli) without any experience of predators (naïve) which could have affected the sub adults’ responses. Alternatively, juvenile lobster have greater mass-specific energy demands and smaller energy reserves (Jensen *et al*., 2013; Simon *et al*., 2015) and thus could be at greater risk of starvation than adults, which could drive greater predator risk-taking behaviours in order to support greater food consumption demands.

Individual activity within a respirometric chamber is metabolically expressed as routine metabolism, and studies have defined the relationship between animal behaviour and metabolic rates (see Toscano and Monaco, 2015). Animals that move more in the chambers consume more oxygen, which has been largely documented in crustacean respiratory physiology (Crear and Forteath, 2000; Kemp *et al*., 2009; Toscano and Monaco, 2015; Kenison and Williams, 2018). This can be further supported by studies examining circadian patterns in activity and their implications for animal metabolic rates (Briceño *et al*., 2018). Toscano and Monaco (2015) found a mismatch between crab activity within a respirometer and that of crabs in a mesocosm when exposed to waterborne cues from predators. While crabs in a mesocosm showed reduced activity, animals within chambers exhibited greater activity (Toscano and Monaco, 2015). Under predation risk, a reduction in prey activity is widely observed in crustaceans (Hazlett, 2011). It has been suggested that increasing oxygen consumption could be due to attempts to hide or escape (i.e. stress) as refuge was not provided within respirometry chambers (Toscano and Monaco, 2015). Conversely, the current study did find that sub adult lobsters decreased their oxygen consumption matching the same behavioural response tested in mesocosms by Toscano and Monaco (2015). The respirometer used here included a shelter inside, probably facilitating the sheltering behaviour of lobsters. Studies testing the immobility response should examine methodological differences in order to better determine the links between behaviour and physiology.

### Escaping responses (tail-flipping) and associated energetic cost

Lobsters did not show differences in AMRs at either temperature, independent of the predation risk scenario. AMR is associated with maximum short-term energy during forced locomotion (Biro and Stamps, 2010), and it is determined by chasing to elicit tail-flipping (e.g. Jensen *et al*., 2013; Fitzgibbon *et al*., 2014). Here, the lobster escape response was similar and was independent of environmental stressors (e.g. warming temperature) and exposure to predator cues. Firstly, the lack of differences in response between temperatures may suggest that sub adults reached maximum active metabolism, probably reaching the thermal limits as previously discussed in regard to the AS. Secondly, animals did not show differences between predation risk levels, indicating that tail-flipping imposed a similar energetic cost independently of predation risk scenario.

While post-exercise oxygen consumption rate (EPOC) was elevated under the warming scenario, it did not recover to pre-exhaustion routine metabolism levels within the first hour of EPOC under both temperatures. Previous research with other rock lobster species (*S. verreauxi*) suggests that the duration and magnitude of EPOC increases with temperature, and the recovery periods after exhaustion may take more than 10 h (Jensen *et al*., 2014; Fitzgibbon *et al*., 2014). This suggests a significant anaerobic capacity of rock lobsters, which demonstrates the large energetic cost associated with tail-flipping. Such anaerobic capacity is associated with large muscle fibres that facilitate tail-flipping as an escape response (Jimenez *et al*., 2008). In an ecological context, a predator attack until exhaustion would impose a large energetic cost beside the risk of death.

Overall, this study demonstrates that for *J. edwardsii* sub adults, temperature can override anti-predator avoidance, such as immobility, under warming scenarios projected for the south-east Australian region. A conceptual model is presented to summarize major findings from this research, highlighting possible foraging behaviour in sub adult *J. edwardsii* (Fig. 7). It is unclear how depletion of key lobster habitats, such as kelp forests, due to the incursion of the habitat-modifying sea urchin (Ling *et al*., 2008; Johnson *et al*., 2011), could challenge the increasing energetic requirements of lobsters under predation risk at warmer temperatures. Lobster juveniles can increase foraging efficiency under predation risk if refuge areas can supply enough food (‘shelter-based food supply’) reducing energetic costs and exposure to predators (Wahle, 1992). However, there are uncertainties in how lobster foraging ecology may be affected as warming temperatures result in reduced habitat quality (e.g. food supply) via urchin barrens (Ling *et al*., 2008). Future studies examining physiological and behavioural responses of lobsters threatened by predators is needed to validate outcomes from this study, especially to understand how changes in physiological and behavioural traits of prey may be reflected at the population level. Major findings reported here can serve as an eco-physiological framework for future studies addressing questions regarding predator–prey interactions in this region, particularly potential impacts for the lobster population, associated fisheries and ecosystem functioning.

## Supplementary Material

Supplementary_information_experimental_set_up_coaa065

Supplementary_Table_1_Protocol_Predation_risk_coaa065

Supplementary_video_coaa065

## References

[ref1a] Anderson TJ (1999) Morphology and biology of Octopus maorum Hutton 1880 in northern New Zealand. Bull Mar Sci 65: 657–676.

[ref1] Angilletta MJ, Wilson RS, Navas C, James RS (2003) Trade-offs and the evolution of thermal reaction norms. Trends Ecol Evol 18: 234–240.

[ref2] Atema J (1995) Chemical signals in the marine environment: dispersal, detection, and temporal signal analysis. PNAS 92: 62–66. 7816848 10.1073/pnas.92.1.62 PMC42817

[ref3] Berger DK, Butler MJ (2001) Octopuses influence den selection by juvenile Caribbean spiny lobster. Mar Freshw Res 52: 1049–1053.

[ref4] Biro P, Stamps J (2010) Do consistent individual differences in metabolic rate promote consistent individual differences in behavior? Trends Ecol Evol 25: 653–659. 20832898 10.1016/j.tree.2010.08.003

[ref6] Briceño, Leόn R, Gardner C, Hobday AJ, André J, Frusher SD, Pecl GT (2015) Spatial variation in mortality by in-pot predation in the Tasmanian rock lobster fishery. Fish Oceanogr. 25: 6–18.

[ref7] Briceño FA, Polymeropoulos ET, Fitzgibbon QP, Dambacher JM, Pecl GT (2018) Changes in metabolic rate of spiny lobster under predation risk. Mar Ecol Prog Ser 598: 71–84.

[ref8] Buscaino G, Filiciotto F, Gristina M, Buffa G, Bellante A, Maccarrone V, Patti B, Mazzola S (2011) Defensive strategies of European spiny lobster *Palinurus elephas* during predator attack. Mar Ecol Prog Ser 423: 143–154.

[ref9] Butler MJ, Lear JA (2009) Habitat-based intraguild predation by caribbean reef octopus *Octopus briareus* on juvenile caribbean spiny lobster *Panulirus argus*. Mar Ecol Prog Ser 386: 115–122.

[ref10] Butler MJ, MacDiarmid AV, Booth JD (1999) The cause and consequence of ontogenetic changes in social aggregation in New Zealand spiny lobsters. Mar Ecol Prog Ser 188: 179–191.

[ref11] Careau V, Killen S, Metcalfe NB (2015) Adding fuel to the “fire of life”: Energy budget across levels of variation in ectotherms and endotherms In Martin LB, Ghalambor CK, Woods HA, eds, Integrative Organismal Biology. John Wiley & Sons, Inc, pp. 219–233.

[ref13] Carter C, Westbury H, Crear B, Simon C, Thomas C (2015) Agonistic behaviour in juvenile southern rock lobster, *Jasus edwardsii* (Decapoda, Palinuridae): implications for developing aquaculture. Zookeys 457: 323–337. 10.3897/zookeys.457.6760 PMC4283379 25561845

[ref14] Childress MJ, Herrnkind WF (1996) The ontogeny of social behaviour among juvenile Caribbean spiny lobsters. Anim Behav 51: 675–687.

[ref14a] Childress MJ, Jury SH (2006) Behaviour In Phillips BF (ed) Lobsters: Biology, Management, Aquaculture and Fisheries. Blackwell Publishing, pp. 78–102.

[ref15] Childress MJ (2007) Comparative sociobiology of spiny lobsters In Evolutionary Ecology of Social and Sexual Systems: Crustaceans as Model Organisms. Oxford University Press, Oxford, pp. 271–293.

[ref16] Chivers DP, Dixson DL, White J, MI MC, MOC F (2013) Degradation of chemical alarm cues and the assessment of risk throughout the day. Ecol Evol 3: 3925–3934. 24198950 10.1002/ece3.760 PMC3810885

[ref17] Cooke SJ, Steinmetz J, Degner JF, Grant EC, Philipp DP (2003) Metabolic fright responses of different-sized largemouth bass (*Micropterus salmoides*) to two avian predators show variations in nonlethal energetic costs. Can J Zool 81: 699–709.

[ref18] Crear BJ, Forteath GNR (2000) The effect of extrinsic and intrinsic factors on oxygen consumption by the southern rock lobster, *Jasus edwardsii*. J Exp Mar Biol Ecol 252: 129–147. 10962070 10.1016/s0022-0981(00)00243-4

[ref19] Culler LE, McPeek MA, Ayres MP (2014) Predation risk shapes thermal physiology of a predaceous damselfly. Oecologia 176: 653–660. 25234372 10.1007/s00442-014-3058-8

[ref20] Dell AI, Pawar S, Savage VM (2014) Temperature dependence of trophic interactions are driven by asymmetry of species responses and foraging strategy. J Anim Ecol 83: 70–84. 23692182 10.1111/1365-2656.12081

[ref21] Díaz ER, Thiel M (2004) Chemical and visual communication dining mate searching in rock shrimp. Biol Bull 206: 134–143. 15198939 10.2307/1543637

[ref22] Draper AM, Weissburg MJ (2019) Impacts of global warming and elevated CO_2_ on sensory behavior in predator-prey interactions: a review and synthesis. Front Ecol Evol 7: 72. doi: 10.3389/fevo.2019.00072.

[ref23] Doubleday ZA, Prowse TAA, Arkhipkin A, Pierce GJ, Semmens J, Steer M, Gillanders BM (2016) Global proliferation of cephalopods. Curr Biol 26: R406–R407. 27218844 10.1016/j.cub.2016.04.002

[ref24] Ferrari MCO, Wisenden BD, Chivers DP (2010) Chemical ecology of predator–prey interactions in aquatic ecosystems: a review and prospectus. Can J Zool 88: 698–724.

[ref25] Fitzgibbon QP, Ruff N, Tracey SR, Battaglene SC (2014) Thermal tolerance of the nektonic puerulus stage of spiny lobsters and implications of ocean warming. Mar Ecol Prog Ser 515: 173–186.

[ref26] Fitzgibbon QP, Simon CJ, Smith GG, Carter CG, Battaglene SC (2017) Temperature dependent growth, feeding, nutritional condition and aerobic metabolism of juvenile spiny lobster, *Sagmariasus verreauxi*. Comp Biochem Physiol Part A: Mol Integr Physiol 207: 13–20. 10.1016/j.cbpa.2017.02.003 28179140

[ref27] Fry FEJ (1947) Effects of the environment on animal activity. Publ Ontario Fish Res Lab 68: 1–52.

[ref28] Fry FEJ (1971) The effect of environmental factors on the physiology of fish In Hoar WS, Randall DJ, eds, Fish Physiology. Academic Press, San Diego, pp. 1–99.

[ref29] Gardner C, Frusher S, Barrett N, Haddon M, Buxton C (2006) Spatial variation in size at onset of maturity of female southern rock lobster *Jasus edwardsii* around Tasmania, Australia. Scientia Marina 70: 423–430.

[ref29a] Harrington JJ, Semmens JM, Gardner C, Frusher SD (2006) Predation of trap-caught southern rock lobsters, Jasus edwardsii (Hutton, 1875), in Tasmanian waters by the Maori octopus, Octopus maorum (Hutton, 1880): Spatial and temporal trends. Fish Res 77: 10–16.

[ref30] Hawlena D, Schmitz OJ (2010) Physiological stress as a fundamental mechanism linking predation to ecosystem functioning. Am Nat 176: 537–556. 20846014 10.1086/656495

[ref31] Hazlett B (2011) Chemical cues and reducing the risk of predation In Breithaupt T, Thiel M, eds, Chemical Communication in Crustaceans. Springer, New York, pp. 356–370.

[ref33] Hinojosa IA, Green BS, Gardner C, Jeffs A (2015) Settlement and early survival of southern rock lobster, Jasus edwardsii, under climate-driven decline of kelp habitats. ICES J Mar Sci 72: i59–i68.

[ref34] Hobday AJ, Pecl GT (2014) Identification of global marine hotspots: sentinels for change and vanguards for adaptation action. Rev Fish Biol Fish 24: 415–425.

[ref35] Holopainen IJ, Aho J, Vornanen M, Huuskonen H (1997) Phenotypic plasticity and predator effects on morphology and physiology of crucian carp in nature and in the laboratory. J Fish Biol 50: 781–798.

[ref36] IPCC (2007) Summary for policymakers. In: Climate Change 2007: The physical science basis In Solomon S, Qin D, Manning M, Chen Z, Marquis M, Averyt KB, Tignor M, Miller HL, eds, Contribution of Working Group I to the Fourth Assessment Report of the Intergovernmental Panel on Climate Change. Cambridge University Press, Cambridge, United Kingdom and New York, NY, USA.

[ref37] Jensen MA, Fitzgibbon QP, Carter CG, Adams LR (2013) Effect of body mass and activity on the metabolic rate and ammonia-N excretion of the spiny lobster *Sagmariasus verreauxi* during ontogeny. Comp Biochem Physiol A Mol Integr Physiol 166: 191–198. 23756212 10.1016/j.cbpa.2013.06.003

[ref38] Jensen MA, Fitzgibbon QP, Carter CG, Adams LR (2014) Recovery periods of cultured spiny lobster, *Sagmariasus verreauxi* juveniles: effects of handling, force feeding, exercising to exhaustion and anaesthesia on oxygen consumption and ammonia-N excretion rates. Aquaculture 410–411: 114–121.

[ref39] Jimenez AG, Locke BR, Kinsey ST (2008) The influence of oxygen and high-energy phosphate diffusion on metabolic scaling in three species of tail-flipping crustaceans. J Exp Biol 211: 3214–3225. 18840655 10.1242/jeb.020677

[ref40] Johnson CR et al. (2011) Climate change cascades: shifts in oceanography, species’ ranges and subtidal marine community dynamics in eastern Tasmania. J Exp Mar Biol Ecol 400: 17–32. doi: 10.1016/j.jembe.2011.02.032.

[ref41] Kenison EK, Williams RN (2018, 2018) Training for translocation: predator conditioning induces behavioral plasticity and physiological changes in captive eastern hellbenders (Cryptobranchus alleganiensis alleganiensis) (Cryptobranchidae, Amphibia). Diversity 10: 13. doi: 10.3390/d10010013.

[ref42] Kemp JOG, Britz PJ, Cockcroft AC (2009) Effect of body size, photophase, feeding and emersion on the oxygen consumption of the east coast rock lobster *Panulirus homarus rubellus*. Aquat Res 40: 833–844.

[ref43] Killen SS, Marras S, Metcalfe NB (2013) Environmental stressors alter relationships between physiology and behaviour. Trends Ecol Evol 28: 651–658. 23756106 10.1016/j.tree.2013.05.005

[ref44] Killen SS, Reid D, Marras S, Domenici P (2015) The interplay between aerobic metabolism and antipredator performance: vigilance is related to recovery rate after exercise. Front Physiol 6: 111. 25914648 10.3389/fphys.2015.00111 PMC4391267

[ref45] Klein ES, Hill SL, Hinke JT, Phillips T, Watters GM (2018) Impacts of rising sea temperature on krill increase risks for predators in the Scotia Sea. PLoS ONE 13: e0191011 10.1371/journal.pone.0191011. 29385153 PMC5791976

[ref46] Krams I, Kivleniece I, Kuusik A, Krama T, Freeberg TM, Mänd R, Vrublevska J, Rantala MJ, Mänd M (2013) Predation selects for low resting metabolic rate and consistent individual differences in anti-predator behavior in a beetle. Act Ethol 16: 163–172.

[ref47] Lima SL, Dill LM (1990) Behavioral decisions made under the risk of predation: a review and prospectus. Can J Zool 68: 619–640.

[ref48] Ling SD (2008) Range expansion of a habitat-modifying species leads to loss of taxonomic diversity: a new and impoverished reef state. Oecologia 156: 883–894. 18481099 10.1007/s00442-008-1043-9

[ref49] Ling SD, Johnson CR, Ridgway K, Hobday AJ, Haddon M (2009) Climate-driven range extension of a sea urchin: inferring future trends by analysis of recent population dynamics. Glob Chang Biol 15: 719–731.

[ref49a] Loflen CL, Hovel KA (2010) Behavioral responses to variable predation risk in the California spiny lobster Panulirus interruptus. Mar Ecol Prog Ser 420: 135–144. 10.3354/meps08850.

[ref49b] MacDiarmid AB, Hickey B, Maller RA (1991) Daily movement patterns of the spiny lobster Jasus edwardsii (Hutton) on a shallow reef in northern New Zealand. J Exp Mar Bio Ecol 147: 185–205. doi: 10.1016/0022-0981(91)90182-V.

[ref50] Marras S, Killen SS, Claireaux G, Domenici P, McKenzie DJ (2011) Behavioural and kinematic components of the fast-start escape response in fish: individual variation and temporal repeatability. J Exp Biol 214: 3102–3110. 21865523 10.1242/jeb.056648

[ref51] Matassa CM, Trussell GC (2014) Effects of predation risk across a latitudinal temperature gradient. Oecologia 177: 775–784. 25433694 10.1007/s00442-014-3156-7

[ref52] Marzloff MP, Melbourne-Thomas J, Hamon KG, Hoshino E, Jennings S, Putten IE van, Pecl GT (2016) Modelling marine community responses to climate-driven species redistribution to guide monitoring and adaptive ecosystem-based management. Glob Chang Biol 22: 2462–2474. 26990671 10.1111/gcb.13285

[ref53] Meuthen D, Ferrari MCO, Lane T, Chivers DP (2019) Predation risk induces age- and sex-specific morphological plastic responses in the fathead minnow. *Pimephales promelas* Scientific Reports 9: 15378. 31653876 10.1038/s41598-019-51591-1 PMC6814781

[ref57] Miller LP, Matassa CM, Trussell GC (2014) Climate change enhances the negative effects of predation risk on an intermediate consumer. Glob Chang Biol 20: 3834–3844. 24947942 10.1111/gcb.12639

[ref55] Mills DJ, Johnson CR, Gardner C (2008) Bias in lobster tethering experiments conducted for selecting low-predation release sites. Mar Ecol Prog Ser 364: 1–13.

[ref55a] Mislan KAS, Babcock RC (2008) On rocky reefs with varying predation pressure and habitat complexity. Mar and Fresh Res 59: 246–253. doi: 10.1071/MF07116.

[ref58] Moltschaniwskyj NA, Carter CG (2010) Protein synthesis, degradation, and retention: mechanisms of indeterminate growth in cephalopods. Physiol Biochem Zool 83: 997–1008. 20979497 10.1086/656387

[ref59] Okuyama T (2015) Metabolic responses to predation risk in a jumping spider. J Zool 297: 9–14.

[ref60] Paul N, Novais SC, Lemos MFL, Kunzmann A (2018) Chemical predator signals induce metabolic suppression in rock goby (*Gobius paganellus*). PLoS ONE 13: e0209286 10.1371/journal.pone.0209286. 30557310 PMC6296658

[ref61] Pecl GT et al. (2009) The East Coast Tasmanian Rock Lobster Fishery – Vulnerability to Climate Change Impacts and Adaptation Response Options. Report to the Department of Climate Change, Australia Commonwealth of Australia Available from: http://www.climatechange.gov.au/sites/climatechange/files/documents/03_2013/rock-lobser-report.pdf

[ref62] Pecl GT et al. (2019) Autonomous adaptation to climate-driven change in marine biodiversity in a global marine hotspot. Ambio 48: 1498–1515. doi: 10.1007/s13280-019-01186-x. 31098878 PMC6883019

[ref63] Pörtner HO (2010) Oxygen- and capacity-limitation of thermal tolerance: a matrix for integrating climate-related stressor effects in marine ecosystems. J Exp Biol 213: 881–893. 20190113 10.1242/jeb.037523

[ref64] Picklington R, Dill LM (1995) Predation on females or males: who pays for bright male traits? Anim. Behav. 49: 1122–1124.

[ref65] Development Core Team R (2014) R: A Language and Environment for Statistical Computing. R Foundation for Statistical Computing, Vienna, Austria ISBN 3-900051-07-0

[ref66] Ramos JE, Pecl GT, Moltschaniwskyj NA, Strugnell JM, León RI, Semmens JM (2014) Body size, growth and life span: implications for the polewards range shift of *Octopus tetricus* in south-eastern Australia. PLoS ONE 9: e103480 10.1371/journal.pone.0103480. 25090250 PMC4121162

[ref67] Ramos JE, Pecl GT, Moltschaniwskyj NA, Souza CA, Strugnell J (2018) Population genetic signatures of a climate change driven marine range extension. Scientific reports 8: 9558. 29934542 10.1038/s41598-018-27351-y PMC6015011

[ref68] Robin JP et al. (2014) Transitions during cephalopod life history. Adv Mar Biol 67: 361–437. 24880797 10.1016/B978-0-12-800287-2.00004-4

[ref69] Rodhouse PG, Pierce GJ, Nichols OC, Sauer WH, Arkhipkin AI, Laptikhovsky VV, Lipinski M, Ramos J, Gras M, Kidokoro H (2014) Environmental effects on cephalopod population dynamics: implications for management of fisheries. Adv Mar Biol 67: 99–233. 24880795 10.1016/B978-0-12-800287-2.00002-0

[ref70] Schmitz OJ, Hawlena D, Trussell GC (2010) Predator control of ecosystem nutrient dynamics. Ecol Lett 13: 1199–1209. 20602626 10.1111/j.1461-0248.2010.01511.x

[ref71] Sih A (1985) Evolution, predator avoidance and unsuccessful predation. Am Nat 1: 153–157.

[ref72] Simon CJ, James PJ (2007) The effect of different holding systems and diets on the performance of spiny lobster juveniles, *Jasus edwardsii* (Hutton, 1875). Aquaculture 266: 166–178.

[ref73] Simon CJ, Fitzgibbon QP, Battison A, Carter CG, Battaglene SC (2015) Bioenergetics of nutrient reserves and metabolism in spiny lobster juveniles *Sagmariasus verreauxi*: predicting nutritional condition from hemolymph biochemistry. Physiol Biochem Zool 88: 266–283. 25860826 10.1086/681000

[ref74] Smith RJF (1989) The response of *Asterropteryx semipunctatus* and *Gnatholepis anjerensis* (Pisces: Gobiidae) to chemical stimuli from injured conspecifics, an alarm response in gobies. Ethology 1989 81: 279–290.

[ref76] Steiner UK, Van Buskirk J (2009) Predator-induced changes in metabolism cannot explain the growth/predation risk tradeoff. PLoS ONE 4: 2–5. 10.1371/journal.pone.0006160 PMC2701611 19582147

[ref77] Thomas C, Crear B, Hart P (2000) The effect of temperature on survival, growth, feeding and metabolic activity of the southern rock lobster, J*asus edwardsii*. Aquaculture 185: 73–84.

[ref78] Toscano BJ, Monaco CJ (2015) Testing for relationships between individual crab behavior and metabolic rate across ecological contexts. Behav Ecol Sociobiol 69: 1343–1351.

[ref79] Uhlmann SS, Broadhurst MK (2013) Mitigating unaccounted fishing mortality from gillnets and traps. Fish Fish 183–229. doi: 10.1111/faf.12049.

[ref80] Wahle RA (1992) Body-size dependent anti-predator mechanisms of the American lobster. Oikos 65: 52–60.

[ref81] Weiss HM, Lozano-Álvarez E, Briones-Fourzán P, (2008) Circadian shelter occupancy patterns and predator-prey interactions of juvenile Caribbean spiny lobsters in a reef lagoon. Mar Biol 153: 953–963. doi: 10.1007/s00227-007-0867-x.

[ref82] Zuur AF, Ieno EN, Walker NJ, Saveliev AA, Smith GA (2009) Mixed Effects Models and Extensions in Ecology with R. Springer-Verlag, New York, pp. 574 ISBN 978-0-387-87457-9

