# Supplementary material for: Temperature alters the physiological response of spiny lobsters under predation risk
Source: Conserv Physiol. 2020 Aug 25;8(1):coaa065. doi: 10.1093/conphys/coaa065 (PMC7439581; doi:10.1093/conphys/coaa065)
Supplement: Supplementary_information_experimental_set_up_coaa065 [file supplementary_information_experimental_set_up_coaa065.pdf]

SUPPLEMENTARY INFORMATION ON EXPERIMENTAL SET-UP

Briceño *et al* 2020 - Temperature alters the physiological response of spiny lobsters under predation risk. Conservation Physiology, doi: 10.1093/conphys/coaa065

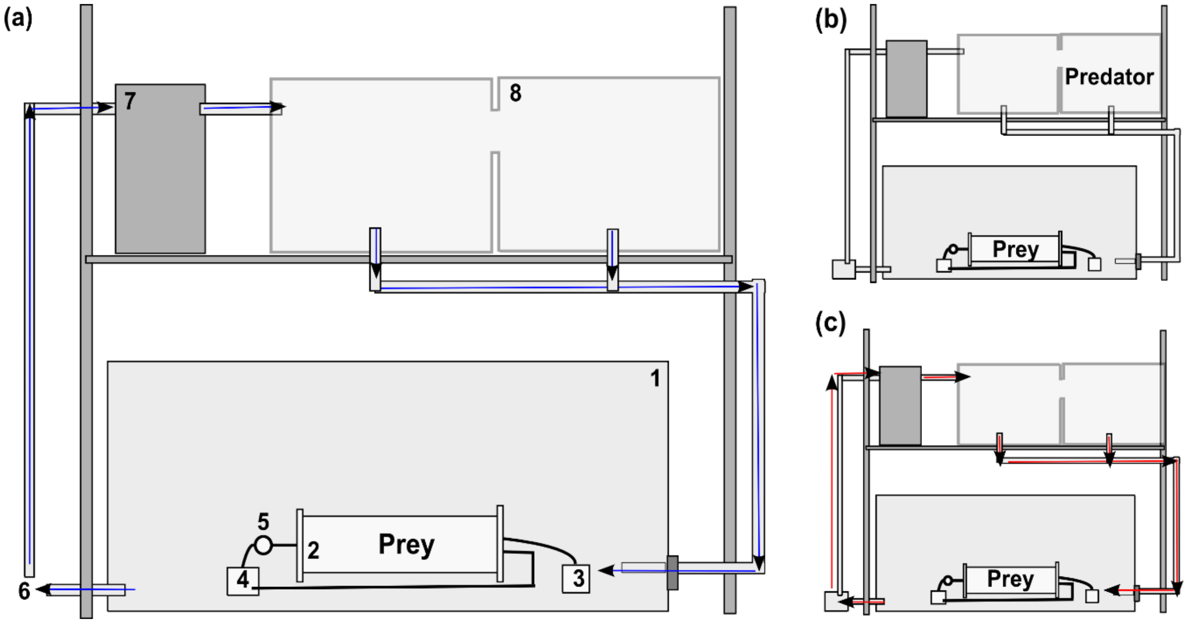

Figure 1: Experimental set-up used on sub-adult Jasus edwardsii (prey) respirometry under exposure of kairomones from octopus odour (predator). The set-up consisted of (1) bath reservoir (treatment tank); (2) two respirometric chambers; (3) flushing pump (which pumps water from the bath into the chambers); (4) mixing pump; (5) oxygen probe; (6) recirculating pump; (7) heater/chiller unit; (8) conditioning tank. Blue arrows show direction of water circulation throughout the system without kairomones. Red arrows show the direction of water with kairomones from the conditioning tank to the bath.

Water was recirculated by a pump (12 HyperFlow Water Rio pump, TAAM Inc, USA) supplying a constant flow rate between the conditioning tank and the bath. The water used for respirometry was previously treated by mechanical filtration (e.g. cartridge, 50 microns). In addition, water temperature was kept stable (at 20°C or at 23°C) by a heater/chiller unit (400 W, TECO, model TK2000, Italy). A submersible pump was connected from the conditioning tank to the heater/chiller unit described above to maintain the experimental temperature. The experimental temperature in the bath was kept constant by a heater (Aqua heat 600 W incl. Nema) during the generation of kairomones. The bath was isolated from the exterior with an opaque plastic sheet (10 mm), keeping the natural light conditions (15–13 hours of day length) to promote the circadian pattern of lobster activity. Additionally, an infrared light was installed above the bath allowing the observation of individuals during night time, in particular to confirm lobster activity before kairomone exposure. Such lighting conditions did not alter *J. edwardsii* circadian patterns as previously reported by Mills et al. (2005). Respirometry was conducted under a natural light cycle, considering the nocturnal period between 20:00 and 8:00.

References

Mills DJ, Verdouw G, Frusher SD (2005) Remote multi-camera system for in situ observations of behaviour and predator/prey interactions of marine benthic macrofauna. NZ J Mar Fresh Res 39: 347–352.
