# Supplementary material for: Temperature alters the physiological response of spiny lobsters under predation risk
Source: Conserv Physiol. 2020 Aug 25;8(1):coaa065. doi: 10.1093/conphys/coaa065 (PMC7439581; doi:10.1093/conphys/coaa065)
Supplement: Supplementary_Table_1_Protocol_Predation_risk_coaa065 [file supplementary_table_1_protocol_predation_risk_coaa065.pdf]

SUPPLEMENTARY TABLE 1: PROTOCOL DEVELOPED TO DEPICT PREDATION RISK SCENARIOS

Briceño *et al* 2020 - Temperature alters the physiological response of spiny lobsters under predation risk.
Conservation Physiology, doi: 10.1093/conphys/coaa065

Step	Description
Step 1: Acclimation in the respirometer	Two lobsters were placed into respirometry chambers following the previous description, being subjected to the same experimental conditions. Animals were acclimated in respirometry chambers for six hours, generally starting around 12:00 pm.<br>Treatment tank and bath are connected through the recirculation pump (Figure 1a)
Step 2: Recirculation pump off	The conditioning tank was separated from the bath by switching off the recirculating pump, immediately before octopus transfer (step 3). Experimental temperature of respirometers was kept as described in methods.
Step 3: Generation of kairomones from octopus	Octopus was taken from the acclimation tank by placing it within a monofilament mesh bag (‘onion bag’), allowing an easier addition into the conditioning tank. Such procedure did not result in a stressful condition as inking or abnormal movement were absent.<br>Octopus remained in the conditioning tank for one hour, generally at midnight.<br>Octopus was gently removed from the conditioning tank and placed back into the acclimation tank.
Step 4: Kairomone exposure	Lobsters were exposed to kairomones between 01:00 and 02:00 during highest activity of individuals in the respirometer. We assumed that this first kairomone exposure was highly concentrated it is considered as an acute exposure.<br>Kairomones were introduced into the respirometric chambers via flushing pumps (see Figure 1c).
Step 5: Recirculation pump on	Both sections (conditioning tank and bath) were re-connected by turning the recirculating pump on (as Figure 1b). The experimental system remained with recirculation until the end of each trial.<br>This assumes that kairomone was diluted over the time until the end of respirometry.
