# Supplementary figures and images for: Temperature alters the physiological response of spiny lobsters under predation risk
Source: Conserv Physiol. 2020 Aug 25;8(1):coaa065. doi: 10.1093/conphys/coaa065 (PMC7439581; doi:10.1093/conphys/coaa065)

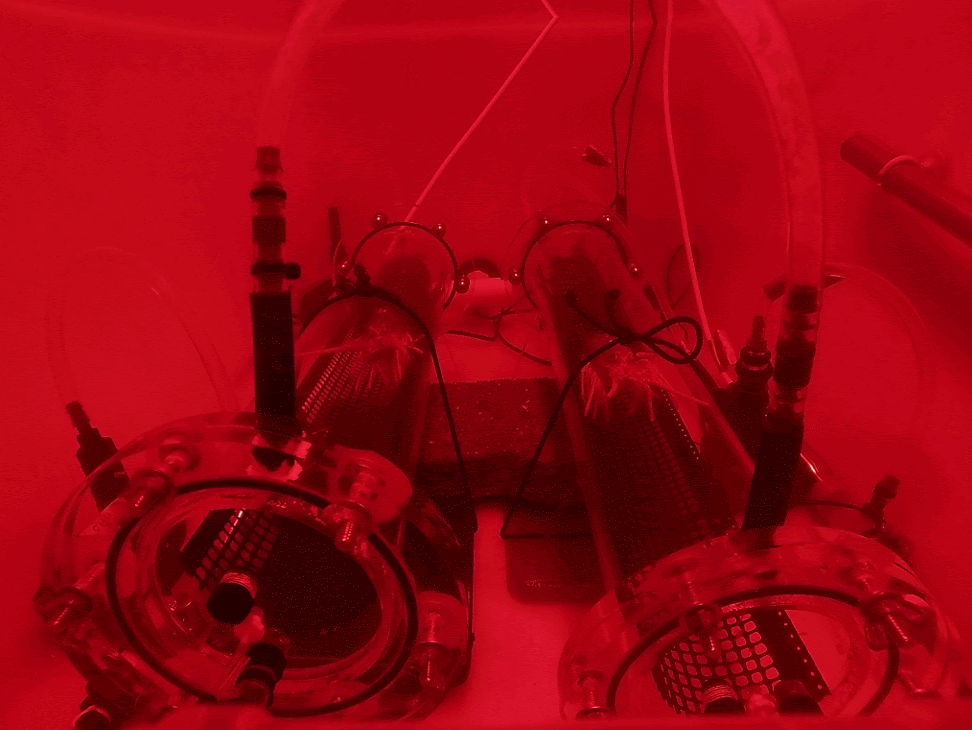

Supplement: Supplementary_video_coaa065 [file supplementary_video_coaa065.zip › Video respirometry_supplementary.gif]
